# CEACAM1 and MICA as novel serum biomarkers in patients with acute and recurrent pericarditis

**DOI:** 10.18632/oncotarget.7530

**Published:** 2016-02-20

**Authors:** Gal Markel, Massimo Imazio, Nira Koren-Morag, Gilli Galore-Haskel, Jacob Schachter, Michal Besser, Davide Cumetti, Silvia Maestroni, Arie Altman, Yehuda Shoenfeld, Antonio Brucato, Yehuda Adler

**Affiliations:** ^1^ Ella Lemelbaum Institute of Melanoma, Sheba Medical Center, Tel Hashomer, Israel; ^2^ Talpiot Medical Leadership Program, Sheba Medical Center, Tel Hashomer, Israel; ^3^ Department of Clinical Microbiology and Immunology, Sackler Faculty of Medicine, Tel Aviv University, Tel Aviv, Israel; ^4^ Cardiology Department, Maria Vittoria Hospital, Torino, Italy; ^5^ Department of Epidemiology, Sackler Faculty of Medicine, Tel Aviv University, Tel Aviv, Israel; ^6^ Internal Medicine, Ospedali Riuniti, Bergamo, Italy; ^7^ Internal Medicine B, Sheba Medical Center, Tel Hashomer, Israel; ^8^ Zabludowicz Center for Autoimmune Diseases, Sheba Medical Center, Tel Hashomer, Israel; ^9^ Cardiac Rehabilitation Institute, Sheba Medical Center, Tel Hashomer, Israel; ^10^ Department of Internal Medicine, Sackler Faculty of Medicine, Tel Aviv University, Tel Aviv, Israel

**Keywords:** pericarditis, serum, biomarkers, MICA, MICB

## Abstract

**Background:**

The immune response plays a significant role in pericarditis, but the mechanisms of disease are poorly defined. Further, efficient monitoring and predictive clinical tools are unavailable. Carcinoembryonic antigen cell adhesion molecule 1 (CEACAM1) is an immune-inhibitory protein, while MHC class I chain related protein A (MICA) and B (MICB) have an immune-stimulating function.

**Methods and results:**

Serum CEACAM1, MICA and MICB concentrations were measured by ELISA in ∼50 subjects of each group: acute pericarditis (AP), recurrent pericarditis (RP) and lupus (SLE) patients, metastatic melanoma patients as well as healthy donors. Serum CEACAM1 was dramatically elevated in AP and RP patients, but not in SLE patients, and displayed a highly accurate profile in ROC curve analyses. MICA and MICB were elevated in some pericarditis patients. All markers were enhanced in metastatic melanoma patients irrespective of neoplastic pericardial involvement. Etiology-guided analysis of RP patients showed that very low MICA levels were associated with idiopathic RP, while high MICA was associated with autoimmune and post-operative RP. Importantly, MICA was significantly associated with recurrences, independently of other potentially confounding parameters such as age, time of follow up or treatment modality.

**Conclusions:**

Here we report for the first time on CEACAM1 as a potentially novel biomarker for pericarditis, as well as on MICA as an innovative prognostic marker in these patients. Determination of the roles of these immune factors, as well as their diagnostic and prognostic values should be determined in future prospective studies.

## INTRODUCTION

Acute pericarditis (AP) is an inflammatory disease of the pericardium, diagnosed in one in every 1000 hospital admissions in the United States [1]. Several etiologies may account for AP, including viral, bacterial, autoimmune, post-pericardiotomy, postmyocardial infarction, cardiac trauma, metabolic and neoplasm [2]. Employing invasive procedures such as pericardioscopy or pericardial biopsy can diagnose the etiology in the vast majority of the cases [2]. Unfortunately, these are not performed in most medical centers on a routine basis. Thus, the etiology in developed countries with a low prevalence of tuberculosis remains unknown (idiopathic) in 85% of the cases. Treatment usually consists of empirical anti-inflammatory therapies, such as non-steroidal anti-inflammatory drug (NSAID), corticosteroids, colchicine and treatment of the underlying cause, when possible [1]. Recurrent pericarditis (RP) is generally manifested by recurrence of AP symptoms after resolution and elimination of the inciting agent [3–5]. RP develops in 30% of AP patients not treated with colchicine [3–7], usually within 18 to 20 months after the initial AP episode, but may occur after longer periods [7, 8]. The disease usually has a relapsing-remitting pattern [3–5], but may be more chronic in some cases [9].

Based on serological findings and frequent responsiveness to immunosuppressive therapy, the immune system seems to play a role in RP. European investigators have recently demonstrated a higher prevalence of infectious etiology (infection or re-infection) by employing pericardioscopy, epicardial biopsy and polymerase chain reaction [9, 10]. Thus, autoreactive pericarditis can be determined only if other etiologies have been excluded and pericardial fluid analysis reveals several immunological features, including increased number of mononuclear cells, anti-sarcolemmal antibodies and inflammatory cytokines (interleukins-6 and 8 and interferon-gamma) [9, 10]. Clinical features have a limited yield in predicting the development of RP, but lack of response to NSAID treatment increases the risk for RP and pericardial constriction [11]. Similarly, inappropriate corticosteroid therapy in AP promotes development of RP, possibly due to enhanced viral replication [6, 11–13]. Up to date, the biological mechanisms for relapses are still elusive, and there are no reliable biomarkers with the capability to predict or monitor development of relapses.

MICA and MICB are encoded in the MHC cluster and share certain homology with HLA class I genes (28–35%) [14]. Normally, MICA and MICB are expressed by intestinal epithelium [15]. However, induction of MICA and MICB expression by a broad spectrum of cells occurs in response to cellular stress, such as transformation, infection and hypoxia [15]. These proteins are ligands for the powerful lymphocyte killer receptor NKG2D [16]. Carcinoembryonic Antigen Cell Adhesion Molecule (CEACAM)-1 is a multifunctional glycoprotein that directly inhibits various effector functions of NK and T cells, such as IFNγ release and killing activities, via homophilic intercellular interactions [17, 18]. This mechanism is exploited by tumor cells [19, 20], viruses [21] and fetal trophoblasts [21].

Here we study these three proteins in the serum of 49 AP patients, 48 RP patients and 47 demographically matched healthy donors, as well as in 50 patients with a known autoimmune disease (SLE) and in 44 metastatic melanoma patients. The potential implications on the immuno-pathogenesis of pericarditis, as well as the potential implementations as innovative clinical tools are discussed.

## RESULTS

### Comparative analysis of inflammatory cytokines

Serum samples were obtained from 49 patients afflicted with acute pericarditis (AP), 48 patients with recurrent episodes of acute pericarditis (RP) and 47 demographically matched controls. These groups were comprised of 29 males and 20 females, 29 males and 19 females, and 27 males and 20 females, respectively (Table 1). There were no significant differences between males and females in all parameters among all experimental groups, including the median follow up time and the number of recurrences in the RP group. The patients were not selected for a certain treatment or inflammatory index (data not shown). SLE and melanoma served as reference diseases. SLE represents a chronic state of autoimmune inflammation, which commonly affects serosal surfaces such as the pericardium. There is no data on soluble CEACAM1, MICA or MICB in SLE. Pericardial involvement by cancer is represented here by melanoma, as 44 metastatic melanoma patients were included, 18 with neoplastic pericardial effusion and 26 without neoplastic pericardial effusion. Serum CEACAM1, MICA and MICB were previously reported as poor prognostic markers in melanoma [22–25], but the relevance of pericardial involvement has never been evaluated.

**Table 1:** Basic clinical data

	Parameter	Males	Females	*P* value	All
Healthy	**N**	27	20	−	47
	**Age (years)**	48	50.4	0.73	49
AP	**N**	29	20	−	49
	**Age (years)**	46.1	48.2	0.65	47
RP	**N**	29	19	−	48
	**Age (years)**	46.2	48.2	0.88	47
	**Follow up (years)**	5.5	4.8	0.42	5.2
	**Recurrences (N)**	5.5	3	0.13	4.5

Table describes demographic data of healthy donors, AP and RP patients, selected clinical parameters and concentration of each of the biomarkers.

**Figure 1:**
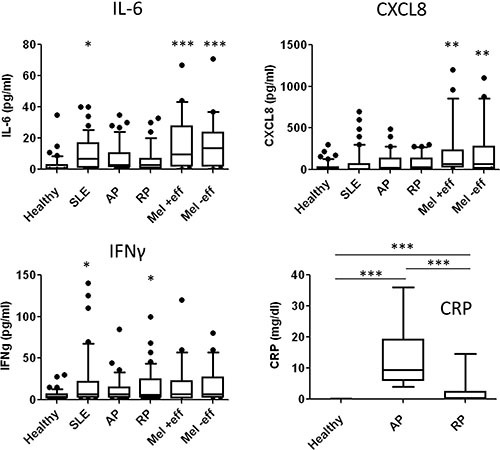
Distribution analysis of inflammatory biomarkers Distribution analysis of each of the indicated biomarkers according to serum concentrations (y-axis) in each group of subjects: healthy donors (Healthy), acute pericarditis patients (AP), recurrent pericarditis patients (RP), systemic lupus erythematosis patients (SLE), metastatic melanoma (Mel) with or without pericardial involvement (+/− eff). Boxes and Whiskers present all data, horizontal line reflects the median value. Statistical significance was tested with Kruskal-Wallis test: ***denotes *P* < 0.0001, **denotes *P* < 0.01 and *denotes *P* < 0.05.

We studied the expression of inflammatory cytokines including IL-6, CXCL8 and IFNγ. In line with previous reports [26], the serum levels of IL-6 and CXCL8 were not significantly elevated among AP and RP patients, as opposed to higher levels observed in metastatic melanoma patients, similar to previously reports [27, 28] (Figure 1). Serum levels of IFNγ were somewhat higher in all of the different patient groups, but statistical significance was demonstrated only among the SLE and RP patients (Figure 1). There were no significant differences between melanoma patients with or without pericardial effusion (Figure 1). As expected, the highest CRP level was observed among the AP patients, but also significantly higher levels than in healthy donors were observed in the RP patients (Figure 1). There were no differences in inflammatory cytokine levels among males and females (data not shown).

As previous reports failed to associate the inflammatory cytokines, which probably emanate from innate immunity components, with certain etiologies or with prognostic parameters, we decided to focus on other novel serum markers, CEACAM1, MICA and MICB. These proteins probably emanate from the injured tissue and have immune regulatory functions, as detailed in the introduction, and therefore might provide new insights on pericardial diseases.

### Characteristics of CEACAM1, MICA and MICB as serum biomarkers in healthy donors

**Figure 2:**
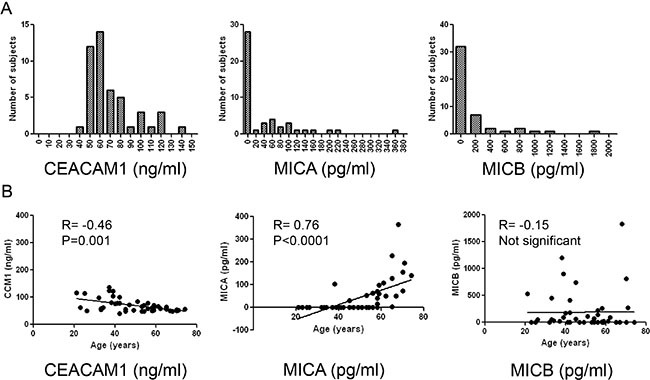
Distribution analysis of biomarkers in healthy donors (**A**) Distribution analysis of each of the indicated biomarkers according to serum concentrations (X-axis). Y-axis denotes the number of patients (frequency); (**B**) Correlation of each biomarker with age among the healthy donors. Correlation was tested with Spearman's test, the *R* and *P* values are indicated in each plot.

Distribution analysis of serum CEACAM1, MICA and MICB levels was carried out in healthy donors. CEACAM1 displayed normal distribution pattern with narrow range and a peak at 60 ng/ml. MICA displayed a relatively narrow range, varying between below threshold of detection to several dozens of pg/ml. MICB demonstrated high variability with an abnormal distribution pattern, ranging from below threshold of detection, through several dozens and hundreds, to several thousands of pg/ml (Figure 2A). A strong correlation was observed between MICA and age (*r* = 0.76, *p* < 0.0001) and an intermediate inverse correlation was observed between CEACAM1 and age (*r* = −0.46, *p* = 0.001). These were further confirmed by a linear regression analysis (*p* < 0.0001 and *p* = 0.0002, respectively). However, these associations occur over a narrow range of values, thus the biological significance of these findings is unclear. MICB did not correlate with age (Figure 2B). There were no significant differences in CEACAM1, MICA and MICB between males and females (data not shown).

### Patients with pericarditis show significantly different pattern of serum CEACAM1, MICA and MICB

**Figure 3:**
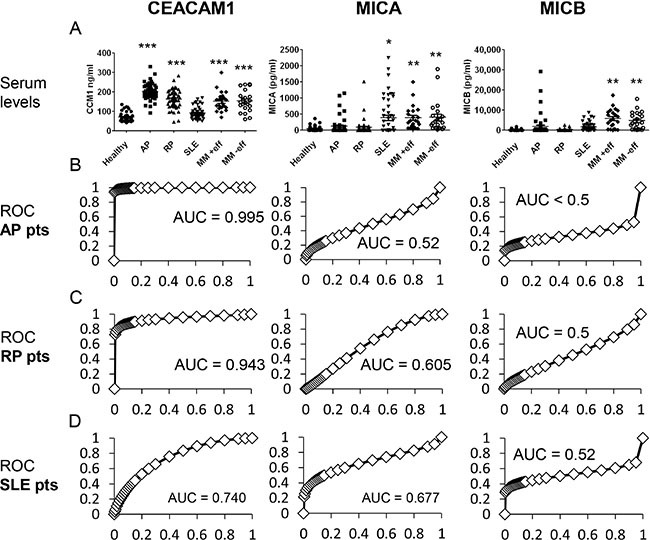
Comparison of biomarker levels between healthy donors and patients (**A**) Serum levels of CEACAM1, MICA and MICB among four subject populations: healthy donors (Healthy), acute pericarditis patients (AP), recurrent pericarditis patients (RP), systemic lupus erythematosis patients (SLE) and metastatic melanoma (MM) with or without pericardial involvement (+/− eff). Each dot represents a patient. Statistical significance was tested with Kruskal-Wallis test: ***denotes *P* < 0.0001, **denotes *P* < 0.01 and *denotes *P* < 0.05; (**B**–**D**) ROC curves of each biomarker (indicate in the top of the Figure) for each group of patients (indicated in the left). Area Under the Curve (AUC) is indicated in each plot.

Serum CEACAM1 levels were markedly elevated in AP patients (2.9-fold, *p* < 0.0001) and in RP patients (2.1-fold in average, *p* < 0.0001), as compared to the healthy donors. Noteworthy, CEACAM1 was significantly elevated in AP patients as compared to RP patients (*p* < 0.001) (Figure 3A). CEACAM1 serum levels in SLE patients (*n* = 50) and healthy donors were similar (Figure 3A). Higher serum MICA levels were observed in AP patients (2.7-fold, *p* = 0.05) and SLE patients (10-fold, *p* < 0.01), as compared to healthy donors and RP patients (Figure 3A). Serum MICB levels were significantly higher in the AP patients (9.6-fold in average, *p* < 0.05) and in SLE patients (7.6-fold in average, *p* < 0.05), as compared to healthy donors and RP patients. In line with previous reports [22, 29], all three markers were detected in significantly higher levels among metastatic melanoma patients. Importantly, there were no differences in these serum markers among melanoma patients with or without neoplastic pericardial effusion (Figure 3A). Noteworthy, the correlations of CEACAM1 and MICA with age observed in healthy donors (Figure 1B) were not evident in the AP and RP patient populations (Table 2A).

**Table 2:** Correlations between serum biomarkers and clinical parameters

A	Age-MICA	Age-MICB	Age-CCM1
**Healthy**	0.760(***)	−0.151	−0.465(**)
**AP**	−0.153	−0.124	−0.108
**RP**	−0.059	−0.301	0.203
**B**	**MICA-MICB**	**MICA-CCM1**	**MICB-CCM1**
**Healthy**	−0.005	−0.256	0.107
**AP**	0.602(***)	0.102	0.104
**RP**	0.380(**)	−0.514(***)	−0.370(*)
**C (RP)**	**Recurrences**	**Age**	**Time**
**Recurrences**	1	−0.326(*)	0.478(**)
**Age**	−0.326(*)	1	0.091
**Time**	0.478(**)	0.091	1
**MICA**	0.306(*)	−0.059	0.1
**MICB**	0.029	*−0.*301	−0.032
**CEACAM1**	−0.167	0.203	0.012

Each of the three biomarkers was correlated with age (A) or with the other biomarkers (B) in healthy, AP and RP patients. In addition, each biomarker was correlated with recurrences, age and duration of follow up (time) in RP patients only (C). Correlation was calculated by Spearman's test. The values in the table represent Spearman's R. *denotes *p* < 0.05, **denotes *p* < 0.01 and ***denotes *p* < 0.001.

ROC curves showed an extremely high accuracy of serum CEACAM1 in pericarditis patients, with AUC values of 0.995 and 0.943 for AP and RP patients, respectively (Figure 3B–3C). In SLE patients, however, the AUC for serum CEACAM1 ROC curve was 0.74 (Figure 3D). ROC curves of MICA and MICB show low accuracy for AP, RP and SLE patients (Figure 3B–3D). ROC curves were not calculated for the melanoma patients, as metastatic malignancy is an entirely different clinical setup than pericarditis, with known association with these three tumor markers.

None of the markers correlated with each other in healthy donors (Table 2B), or with any of the inflammatory cytokines tested, IFNγ or IL-6 (data not shown). In AP patients, a strong correlation between MICA and MICB was observed (*r* = 0.602, *p* < 0.0001), while CEACAM1 did not correlate with MICA or MICB (Table 2B). In RP patients, the correlation between MICA and MICB was weaker but still statistically significant (*r* = 0.38, *P* < 0.001). Interestingly, CEACAM1 was inversely correlated with both MICA (*r* = −0.514, *p* < 0.0001) and MICB (*r* = −0.37, *p* < 0.05) (Table 2B). Noteworthy, the absolute concentration levels of these markers, except for CEACAM1, were not significantly different between the AP or RP patient populations and the healthy donors (Figure 3), only their respective associations with each other among the patients were. These correlations could imply on common regulation mechanisms that might be linked to the underlying pathology, but this is still mostly unclear.

Expectedly, a direct correlation (*r* = 0.478, *p* < 0.01) was observed between recurrences and time of follow-up for all patients (Table 2C). An inverse correlation (*r* = −0.326, *p* < 0.05) was observed between recurrences and age (Table 2C), indicating on a tendency to develop RP at younger age. Finally, and most importantly, a solid correlation (*r* = 0.306, *p* < 0.05) between MICA and recurrences was observed (Table 2C). No similar correlation could be observed with MICB or CEACAM1 (Table 2C). None of the inflammatory markers presented in Figure 1 demonstrated a similar correlation (data not shown).

### Linkage between serum MICA and etiology of recurrent pericarditis

Our cohort of RP patients included 33 “idiopathic” patients, 7 patients with autoimmune pericarditis, 6 “post pericardiotomy” and 2 “post myocardial infarction” (Table 3). Remarkably low serum concentrations of MICA were observed in idiopathic RP patients, as compared to other etiologies of RP (Table 3, *P* value = 0.009). CEACAM1 levels in idiopathic RP patients were the highest, but this was not of statistical significance (Table 3, *P* value = 0.188). Although some differences were observed in serum MICB among different etiological groups, these were not statistically significant (Table 3).

**Table 3:** Correlations between serum biomarkers and RP etiology

Etiology	N	Median MICA	Median MICB	Median CEACAM1
		(pg/ml)	(pg/ml)	(ng/ml)
**Idiopathic**	**33**	3	0	171
**Autoimmune**	**7**	65	0	151
**Post pericardiotomy**	**6**	118	317	130
**Post myocardial infarction**	**2**	179	1223	133
***P* value**		**0.009**	*0.466*	0.188
**Chi square**				

Patients were categorized into four main etiological groups, as indicated in the table. The median values of the biomarkers for each etiological group are presented. Statistical significance was tested with Kruskal-Wallis test.

**Figure 4:**
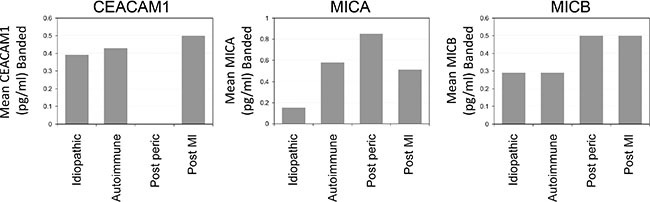
Distribution of RP patients exhibiting high values of biomarkers according to etiological groups Figure shows the percentage of RP patients exhibiting biomarker values in the highest tertile. Patients are categorized in each of the indicated etiological groups.

The percentage of patients exhibiting biomarker values in the highest tertile was calculated. Remarkably, only 15% of the idiopathic RP patients exhibited high MICA levels, as compared to a robust 83% of post pericardiotomy RP patients and 57% of autoimmune RP (Figure 4). These results are in accordance to the statistical link demonstrated above (Table 4). Approximately a third of the idiopathic and autoimmune RP patients, and 50% of the post pericardiotomy and post myocardial infarction RP patients exhibited high values of MICB (Figure 4). Significantly, none of the post pericardiotomy RP patients exhibited high CEACAM1 values, as compared to 39% and 43% of the idiopathic and autoimmune patients, respectively (Figure 4). These results are in accordance with the statistical trend demonstrated above (Table 3). None of the inflammatory markers presented in Figure 1 had any correlation with etiology (data not shown).

**Table 4:** Association between treatment and serum biomarkers

	N	MICA (pg/ml)	MICB (pg/ml)	CEACAM1 (ng/ml)	Recurrences (median)
**All treatments**	5	110	0	124	7.0
**Colchicine + NSAID**	13	3	138	158	3.0
**Colchicine + steroids**	20	3	0	188	3.5
**No Colchicine**	4	53	140	156	8.0
**Only Colchicine**	6	19	92	151	4.5
***P* value**		0.10	0.63	0.12	0.03

Patients were categorized according to treatment combinations as indicated in the table. Biomarker and recurrence values presented are the median value of the group. Statistical significance was tested with Kruskal-Wallis test.

These results imply that RP etiology is associated with differential regulation of stress response proteins. Furthermore, this is the first time that an easily measurable serum marker (MICA) can potentially discriminate between idiopathic RP and other causes of RP.

### Serum MICA correlates with recurrences in RP patients irrespectively of treatment regimen

The potential effect of various treatment regimens on the tested serum biomarkers values was evaluated in the RP patients. Treatment categories included colchicine only, colchicine and corticosteroids, colchicine and NSAIDs, and no colchicine or all (Table 4). At the time of sampling, 26 patients did not receive any treatment, 8 were on colchicine only, 5 on all three modalities, 4 on steroids + colchicine, 2 on NASAIDs + colchicine, 2 on steroids only and 1 on NSAIDs only. An improved clinical outcome of patients treated with colchicine and NSAIDs, or with colchicine and steroids, was observed, as these patients experienced significantly less recurrences (Table 4). Importantly, a clear trend towards very low MICA values was observed in these patients (Table 4, *P* value = 0.10). This strongly implies that serum MICA concentration reflects number of recurrences and is independent of the treatment regimen (Table 4). In addition, a similar trend for higher CEACAM1 values was observed in the same treatment groups (Table 4, *P* value = 0.12). This finding supports the inverse correlation between MICA and CEACAM1 depicted above (Table 2). Despite the apparent differences in MICB values, these were not statistically significant (Table 4, *P* value = 0.63). There were no correlations between any of the serum biomarkers and severe adverse effects of colchicine mandating treatment discontinuation (data not shown).

## DISCUSSION

Pericarditis (acute and chronic) is an inflammatory disorder with several identified etiologies [1, 3, 5]. The specific etiology usually remains unknown as diagnostic invasive procedures are not commonly performed. A substantial portion of these cases is most probably caused by viral infections [9, 10]. Currently, the immuno-pathogenesis of pericarditis is still mostly unknown, and there are no accessible predictive biomarkers. The lack of reliable clinical tools for diagnosis, monitoring and prediction of RP is a withstanding clinical deficit. Few studies on the diagnostic potential of cardiac Troponin I in viral acute pericarditis [30], pericardial fluid adenosine deaminase for tuberculous RP [31, 32] and classical tumor markers like CEA for neoplastic RP [33] were published. Here we investigate the involvement of serum CEACAM1, MICA and MICB, proteins that can be induced in damaged or inflamed tissues, in pericarditis patients.

Cell surface expression of CEACAM1 is induced by viral infection [21] and inflammatory cytokines such as IFNγ [20]. MICA and MICB are induced by similar conditions [15]. Specifically in the heart, it was reported that CEACAM1 expression is induced upon hypoxic injury [34] and that tissue-expressed MICA and MICB were implicated in allograft rejection [35]. As some of these conditions may play a role in pericarditis, upregulation of CEACAM1, MICA or MICB could be anticipated *in situ*. It has been demonstrated in several reports that MICA or MICB proteins are shed by tumor cells in many types of cancers with potential prognostic value [36–38]. Remarkably, it was recently reported that in multiple sclerosis (MS) patients, serum MICB levels are increased and are associated with relapse [39]. In addition, it has recently been shown that the presence of soluble CEACAM1 in the serum reflects tumor burden in pancreatic adenocarcinoma patients [40] and may have prognostic importance in melanoma patients [22]. Collectively, these prompted the investigation of serum CEACAM1, MICA and MICB in pericarditis patients.

We show high serum CEACAM1 levels and efficient ROC curves in AP and RP patients (Figure 3). The underlying biochemical mechanism is still elusive, as it could be due to unique splicing in the injured pericardial cells, like we've previously suggested for melanoma cells [22], or due to other mechanisms such as proteolytic cleavage. The high serum CEACAM1 levels observed in metastatic melanoma patients is irrespective of neoplastic pericardial involvement (Figure 3), implying that different patho-physiological mechanisms account for the enhanced serum CEACAM1 in pericarditis patients. Following this line, serum CEACAM1 concentrations were not elevated in patients with an autoimmune disease like SLE (Figure 3), suggesting on disease specific mechanisms among inflammatory conditions. Further mechanistic studies are mandated, including a biochemical analysis of the serum CEACAM1 in the pericarditis patients. Clinically, the evidence point on a potential diagnostic relevance of serum CEACAM1 in pericarditis patients, which should be further explored in larger prospective clinical trials.

Serum MICA and MICB levels were elevated in some of the pericarditis patients, but without reaching statistical significance or efficient ROC curves (Figure 3). MICA and MICB were strongly elevated in SLE patients and in patients with metastatic melanoma, irrespective of neoplastic pericardial involvement (Figure 3). This argues against a significant diagnostic role for MICA and MICB in pericarditis. Nevertheless, among RP patients, a significant positive correlation between serum MICA concentrations with recurrences was observed, independently of other potentially confounding parameters linked to recurrences, such as age and time of follow up (Table 2), or the therapeutic regimen (Table 4). In normal donors, MICA positively correlates with age (Figure 2), a correlation that is lost in pericarditis patients (Table 2). This indicates on a prognostic value of MICA. An extended group of subjects is required to conduct a prospective multivariate analysis to evaluate the full predictive prognostic potential of MICA in RP patients.

The elevation in MIC proteins could be explained either by systemic spillage from the site of inflammation or from other reactive remote sites, created by protein cleavage by metalloproteinases or secretion of soluble MIC forms [41]. Elevated serum MICA and MICB were reported in cancer and autoimmune conditions [36–38, 42, 43]. It was also reported that MICB is upregulated within active MS lesions, which may be the source for elevated serum MICB in active MS patients [39]. Our findings imply on a potential role for MICA in pericarditis, as it is associated with recurrence. Increased levels of serum MICA cause systemic downregulation of NKG2D expression [42, 44]. Theoretically, this may result in increased susceptibility to viral infection or re-activation, directly leading to recurrences. Indeed, it has been demonstrated more than 2 decades ago that NK cell cytotoxic activity *in vitro* is decreased in perimyocarditis patients [45]. Mechanistic studies on pericardial specimens, epicardial fluids and peripheral blood lymphocytes are thus mandated.

## MATERIALS AND METHODS

### Patients

We enrolled all consecutive patients with acute and recurrent pericarditis seen at Niguarda Hospital, Milan, since 1998 to 2004, who gave written informed consent and whose sera was available for analysis, stored at −20°C. Inclusion criteria were: definite diagnosis of acute or recurrent pericarditis (idiopathic, viral, and autoimmune etiologies including post-pericardiotomy syndromes, and connective tissue diseases), age ≥ 18 years. Exclusion criteria were: bacterial (tuberculous, purulent), and neoplastic etiologies. The clinical diagnosis of recurrence was based at least on recurrent chest pain and one or more of the following signs: fever, pericardial friction rub, electrocardiographic changes, echocardiographic evidence of new or worsening pericardial effusion. Elevation of C-reactive protein or increased erythrocyte sedimentation rate was required in all cases to confirm the diagnosis of recurrence. Age and sex-matched controls were included from healthy blood donors recruited in the same time interval. Serum samples of diagnosed SLE patients, who gave their informed consent, were collected in Sheba Medical Center between the years 2004–2009. Serum from metastatic melanoma patients, who gave their written informed consent, was collected between the years 2006–2014 (IRB approval 847111). Pericardial effusion was primarily documented by disease evaluation CT scans.

### ELISA

Soluble MICA, MICB, IL-6, CXCL8 (IL-8), and IFNγ were quantified in serum samples by using commercial sandwich ELISA kits according to manufacturer's instructions (R & D Systems). Customized CEACAM1 sandwich ELISA was performed as previously described [22], based on an in-house developed mAb to CEACAM1 [19]. Each sample was tested twice in triplicates. CRP was determined by the hospital's accredited clinical automated routine lab.

### Statistical analysis

All statistical analyses were performed with SPSS ver. 14.0 or with Prism GraphPad ver. 5.

The distributions of the biomarkers were examined using the Kolmogorov-Smirnov non-parametric test. Kruskal-Wallis test was used to test: a) the differences in various biomarkers between healthy donors, AP, RP, SLE and melanoma patients; b) the differences in various biomarkers among different etiologies and treatments in the RP group. The association between biomarker values and etiology was further analyzed by dichotomous cutoff: biomarker values below and above the highest tertile. ROC curves were established for each biomarker and the AUC values were calculated. Spearman's test was used to examine the correlations in a multi-parameter matrix comprised of CEACAM1, MICA, MICB, age, recurrences and time of follow up. The significance levels were set at 0.05.
